# Beyond Host Defense: Deregulation of *Drosophila* Immunity and Age-Dependent Neurodegeneration

**DOI:** 10.3389/fimmu.2020.01574

**Published:** 2020-07-22

**Authors:** Srishti Arora, Petros Ligoxygakis

**Affiliations:** Laboratory of Cell Biology, Development and Genetics, Department of Biochemistry, University of Oxford, Oxford, United Kingdom

**Keywords:** aging, drosophila, neurodegeneration, immunity, immunotherapy

## Abstract

Age-dependent neurodegenerative disorders are a set of diseases that affect millions of individuals worldwide. Apart from a small subset that are the result of well-defined inherited autosomal dominant gene mutations (e.g., those encoding the β-amyloid precursor protein and presenilins), our understanding of the genetic network that underscores their pathology, remains scarce. Genome-wide association studies (GWAS) especially in Alzheimer's disease patients and research in Parkinson's disease have implicated inflammation and the innate immune response as risk factors. However, even if GWAS etiology points toward innate immunity, untangling cause, and consequence is a challenging task. Specifically, it is not clear whether predisposition to de-regulated immunity causes an inadequate response to protein aggregation (such as amyloid or α-synuclein) or is the direct cause of this aggregation. Given the evolutionary conservation of the innate immune response in *Drosophila* and humans, unraveling whether hyperactive immune response in glia have a protective or pathological role in the brain could be a potential strategy in combating age-related neurological diseases.

## Introduction

Aging is characterized by the time-dependent deterioration of cellular function and fitness of an organism, accompanied by an increased susceptibility to diseases (1). This decline in function is inexorable, and is a key risk factor for a number of human disease pathologies such as diabetes, cancer, cardiovascular disorders, and neurodegenerative diseases (2). As the world's geriatric population continues to grow at an exceptional rate (3), a substantial economic burden is placed on the healthcare system to deal with the development of age related diseases. Therefore, understanding the mechanism of longevity and identifying targets to improve health during aging is of paramount importance.

Over the last decade there have been a number of attempts to explain the phenomenon of aging (1). Different processes that affect aging can be categorized into nine hallmarks that are shared by aging and age-related diseases (4). These include: altered intercellular communication, stem cell exhaustion, cellular senescence, mitochondrial dysfunction, deregulated nutrient sensing, genomic instability, telomere attrition, loss of proteostasis (protein homeostasis), and epigenetic alterations. These co-occur as an organism ages and are extensively interconnected. However, there are several questions regarding the interconnectedness of these hallmarks. One such question is the role of inflammation and its impact on age-related disorders (5). There is an age-dependent decline in immune response, this phenomenon is termed as “immunosenescence” (6, 7). This process is marked by a reduction in the adaptive immune response and was initially believed to be a consequence of a progressive rise in low-grade chronic pro inflammatory status known as “inflammageing” (8–10). More precisely, as the thymus releases the last pool of naïve T-cells and that pool gets depleted each time there is an infection, older people (>65) become progressively weaker in their T-cell responses (5–7). Increase in innate immune pro-inflammatory levels has been seen as a “balancing act” to counter adaptive response reduction. Nevertheless, recent studies have observed that the two processes are mutually maintained and affect one another (11). For instance, depletion of adaptive immune cells strengthens the innate immune response causing inflammageing; similarly, the increased innate immune inflammatory mediators leads to a reduction in the number of adaptive immune cells causing immunosenescence. Cumulative studies have also indicated an age-dependent change in the innate immune cell types that leads to an overall decrease in their ability to collaborate in the initiation of the adaptive immune response [reviewed by (6)]. Inflammageing is often a result of non-resolving or sterile inflammation and is thought to be as a possible underlying basis for most age related diseases such as infections, cancer, autoimmune disorders, and chronic inflammatory diseases (12). Additionally, cell senescence in other tissues generates cytokines that signal the necessity for these cells to be removed by macrophages, to avoid what is called “by-stander senescence” propagating in tissues. This further enhances age-dependent systemic pro-inflammatory activity (5–9). The above, happens to everyone in the context of healthy aging. Some however, are “high responders” and develop neurodegeneration. Does predisposition to different levels of immunity influence this? This review focuses on the role of immunity in neurodegenerative disorders. The primary goal is to understand the importance of immune regulation and its role in aging and age-related diseases. We start by exploring the correlation between immunity and neurodegeneration connected to specific disorders. Then we explore the advantages and disadvantages of non-vertebrate animal models in studying aging and neurodegeneration. We continue with a brief introduction to *Drosophila* immune response and central nervous system. Finally, we conclude with studies suggesting a role for the immune system as a modulator of basal levels of age-dependent neurodegeneration and talk about the need to explore the potential role of negative regulators in immunotherapy.

## Immunity and Neurodegeneration

While the correlation between inflammation and neurodegeneration is well-known (13), whether inflammation is one of its causes or a consequence remains unclear. Inflammation can be triggered as a *consequence* through the production of apoptotic factors and cytokines signaling during neuronal death However, immune cells produce neurotoxic cytokines that could *cause* death of neurons (14). Initially, the activation of the immune response in the central nervous system (CNS) was believed to be responsible for the elimination of infectious agents and the clearing of debris after injury, suggesting a neuroprotective role of inflammation. A positive role of antimicrobial peptide (AMP) production and aging has been suggested by Loch et al. (15). Genome wide association studies (GWAS) have reported the activation of numerous genes of the inflammatory pathway during aging (16). Age is the greatest risk factor for neurodegenerative disorders and age-related chronic activation of the immune response is a shared feature among many neurodegenerative disorders (17). However, the cause of this sterile inflammation is still unknown.

Studies in animal models indicate the importance of inflammation in several neurodegenerative disease pathologies (18). Altering expression of Cdk5 protein kinase (Cdk5α) leads to disruption in autophagy that in turn leads to upregulation of AMP and age-dependent degeneration of dopamine neurons in *Drosophila* (19). Neuroinflammation has been a crucial factor for the pathogenesis of diseases such as Alzheimer's Disease (AD) (20). Microglia, the resident innate immune cells of the CNS are shown to be chronically activated around these plaques. It is believed that the uncontrolled inflammation of these cells leads to the secretion of multiple neurotoxic factors such as inflammatory mediators and reactive oxygen species by glial cells that aggravate the pathology of the disease (14). It is further demonstrated that mutations in microglial protein TREM2, PLCG2, and ABI3 increase the risk for AD (21). Additionally, molecular and pathological interaction studies have established glial expression of TREM2/TYROBP as a key factor in tau mediated neurodegeneration (22). Activated microglia is suggested as a potential marker to detect AD before the appearance of plaques (23). Additional risk genes for late onset AD connected to microglia and immunity have been identified recently (24). Genetic analysis of these late-onset AD risk genes identified a transcriptional network of 12 largely microglial genes that form a transcriptional network (25). Six of these (*OAS1, LAPTM5, ITGAM, ABI3, PLCG2, SPI1*) have good *Drosophila* homologs expressed in the nervous system (our unpublished observations).

*Drosophila* models illustrate the importance of the Toll mediated NF-κB response in the neurotoxicity cause by the presence of Aβ42, an isoform of the beta amyloid protein. Down regulation of this immune pathway was shown to reduce the pathological activity of Aβ42 (26). Evidence in both human and animal model studies have illustrated the correlation between inflammation and Parkinson's disease (PD) (27). Mechanisms of neuronal dysfunction such as mitochondrial dysfunction and oxidative stress have been linked to pathogenesis of PD (28). Dopaminergic neurons (DA) in the midbrain are shown to be sensitive to pro inflammatory cytokines, reactive oxygen species, and chemokines such as TNF-α and IFN that exacerbating neuronal lesions (29). Additionally, there is a rich population of microglia in the substantia nigra, which is the region of the brain that shows the most DA neuron loss in PD patients (30). Studies have observed correlation between deposition microglial activation and alpha-synuclein making microglia an attractive therapeutic target (31, 32). However, PD is considered as a condition that is hypothesized to starts in the intestine as chronic inflammation, then may transfer α-synuclein to the brain through the vagus nerve (33).

Transgenic mice lacking the TNF- receptor demonstrate a reduction in the TH-immunoreactivity after being exposed to MPTP (1-methyl-4-phenyl-1,2,3,6-tetrahydropyridin). Prolonged use of non-steroidal anti-inflammatory drugs such as ibuprofen is shown to reduce risk of PD (27). Consistent with these studies, an increase in circulating cytokines and increased microglial activation have also been linked to early stages of Huntington's disease (34, 35). However, production of pro inflammatory molecules is not just limited to microglia. Other types of glia cells such as astrocytes are also used to investigate the progression of inherited ALS (36). Studying these processes in humans is extremely challenging. Therefore, to explore the processes that govern aging requires accessible model systems that help provide critical insights into the cellular and molecular levels of aging.

## Animal Models of Aging

There are several challenges in studying primate subjects in aging research. These include a number of ethical issues, environmental factors, as well as their relatively long lifespan. Consequently, aging researchers have turned to unicellular or small animal models to investigate the genetic and physiological mechanisms related to human aging and longevity. These models allow us to better control for several intrinsic and extrinsic factors such as uniformity in background genetics, large sample sizes, genetic tractability as well as environmental factors such as managed nutrient availability and chemically-defined diets. These conditions make mechanistic analysis easier and ultimately help identify novel pharmaceutical targets. Some of the popular models used in aging research include: the budding yeast (*Saccharomyces cerevisiae*), the nematode worm (*Caenorhabditis elegans*), the fruit fly (*Drosophila melanogaster*), and mouse (*Mus musculus*).

### Saccharomyces cerevisiae

Studies in model organism have identified conserved pathways that influence the rate of aging (37). The simplest organism that can be used to study eukaryotic aging is Brewer's yeast or *Saccharomyces cerevisiae*. This single celled living organism shares a number of genes with humans, out of which a significant number carry out the same function in both organisms (38). This includes mechanisms that facilitate pathogen recognition during its vegetative development (18). Fungi possess a class of cytosolic NOD (Nucleotide Oligomerization Domain)- like receptors or NLRs are responsible for self and non-self-recognition. These fungal receptors share homology with the effector domains of several plant or animal NLRs and provide a unique opportunity to explore infectious host-pathogen interactions (39).

Aging in yeast can be studied using two different models. The first, replicative lifespan (RLS) that describes the total number of cell divisions a single mother (virgin) cell undergoes, the second, the chronological lifespan (CLS) that represents the length of time a cell can stay viable in a post mitotic state (40). CLS shows an elevation in DNA damage that is a characteristic that resembles that of post mitotic cellular aging in humans (41). However, both models rely on nutrient availability and negatively affect each other (42). The small genomic size and rapid generation time of 3 h makes yeast a great model for high throughput screening and exploiting genetic interactions that are thought to be involved in human aging (42). It is also used to study the effects of dietary restriction (43), oxidative stress (44), and target of rapamycin (TOR) nutrient response pathway on age related phenotypes (42, 45) Moreover, yeast models are used to study a number of age related diseases including Werner syndrome, (41) Huntington's disease (46), Alzheimer's disease (47), Parkinson's disease (48). The lessons from yeast have given us valuable insights into how stress and aging are modulated in higher organisms. However, yeast lacks the complexity of a higher eukaryotic cell and intercellular interactions that are of major importance in aging and age-related disorders.

### Caenorhabditis elegans

Another invaluable model system to study aging is the small nematode worm, *Caenorhabditis elegans. C. elegans* have a short lifespan of about 2–3 weeks at 20°C. This small worm grows to be about a millimeter in length and displays complex behavior such as avoidance behavior when exposed to pathogens. It is compatible with a wide range of genetic techniques including chemical mutagenesis screens, CRISPR, and RNAi. Unlike yeast, it allows us to study tissue-to-tissue communication by tissue-specific transgenic expression and knockdown techniques in a multicellular context. *C. elegans* lack an adaptive immune response and are devoid of any migratory innate immune cells. Instead the protective immune response relies on three lines of defense. The first is avoidance behavior in which the worm can discriminate between different species of bacteria by recognizing odors of specie specific molecules such as cyclic pentadepsipeptide biosurfactant serrawettin W2 produced by *Serratia marcescens* (49). The second line of defense consists of physical barriers. The strong exoskeleton of *C. elegans* is made up of collagen and chitin that creates a physical barrier limiting the entry of potential pathogens. Additionally, a pharyngeal grinder prevents pathogens from accessing the intestines. The third and final line of defense is the humoral response which involves the activation of conserved signaling pathways (including MAP kinase cascades) that leads to the production of several antimicrobial peptides (50, 51).

*C. elegans* allow us to experimentally demonstrate the roles of several other conserved processes in aging such as caloric restriction, mitochondrial pathways energy metabolism, endocrine signaling, and signal transduction, the stress response, protein translation, and gene expression in aging. (52). At the convergence of immunity and aging, recent studies have shown the role of innate immunity regulated by p38 signaling and the transcription factor ATF-7 as responsible for the lifespan extension caused during dietary restriction (53). However, *C. elegans* are evolutionarily distant from humans and has a very different nervous system organization of just 302 neurons leading to behaviors unique to its lifestyle (54). Nevertheless, studies of neuronal cell death in worms has implicated proteins very closely related to mammalian calpains and cathepsins (55). Calpains are a family of calcium regulated cysteine proteases that are highly expressed in neurons. They affect a wide range of cellular functions including cell division, proliferation, migration, and death. In neurons, these proteases have been linked to synaptic plasticity and neurodegeneration (56). The calpain mediated cleavage of carbonylated Hsp70.1 due to oxidative stress leads to loss of lysosomal integrity and rupture. Among the contents of the lysosome, a hydrolytic enzyme (cathepsins) is also released that takes over the role of a “death-executing proteases” by degrading several cellular proteins (55, 57). This Calpain- mediated cleavage of Hsp70.1 helps elucidate the importance of proteolysis in neuronal death and serves as a promising target for preventive interventions of neuronal death (57). Thus, at the cellular level, worm genetics can provide new insights into brain cell death.

### Mus musculus

While yeast and worms have broadened our understanding into the cellular mechanisms of aging and age-dependent neurodegeneration, they fail to replicate system level neurological changes that occur during human aging. Therefore, mammalian model organisms are essential to unravel these complex mechanisms. What makes the mouse an indispensable model is the easy genetic manipulation, short lifespan, low-cost (compared to other primate models), and considerable similarities with human physiological and cellular function (58). Many mouse models of human aging have been developed and characterized, including models for Werner syndrome (59), Ataxia telangiectasia (60), Alzheimer's (61), and Parkinson's diseases (62). Although the degree of complexity of the mouse brain is lower than that of a human, there are several cellular similarities of the nervous system. They show complex behaviors and are a good tool to measure cognitive changes in neurodegenerative disorders (63).

Studies in mice with Alzheimer's disease have highlighted rapamycin as a valid therapeutic approach for prevention or treatment of AD (64, 65). Mouse models that display lifespan extension and rise in delayed aging phenotypes are in line with the observation that DNA metabolism influences aging. Furthermore, factors such as caloric restriction and defects in genome maintenance have been investigated in mice (66). Li et al. demonstrated the role of chronic high fat diet in mice causes loss of neuronal stem cells in the hypothalamus via IKKβ/NF-κB activation that eventually leads to obesity and pre-diabetes (67). The hypothalamus the neuroendocrine functional center of the body is also responsible for systemic aging though NF-κB signaling (25) and thus provide a potential therapeutic approach to combat age and age related disorders (68). This seems to be an evolutionary conserved component of aging as NF-κB in the brain is a major life span determinant in *Drosophila* as well [(41), see below]. Even though laboratory mice are an admirable model to study some age- related phenotypes, they do not fully mimic the mechanism. Laboratory mice are inbred and age very quickly. They invest more in reproduction and less in somatic maintenance and therefore do not display the trade-off between fecundity and longevity observed in humans. They also do not recapitulate age related disease pathologies as seen in humans (e.g., Werner diseases models and amyloid plaques in AD mice models).

### Drosophila melanogaster

Over the last two decades, *Drosophila* has developed as a powerful tool to investigate human disease mechanisms. It has orthologs of ~65% of all genes causing heritable diseases in humans (69, 70), making it an attractive model organism to address novel lines of inquiry for human diseases (71). Moreover, for every one of these genes, the fly will most of times have one copy while humans will normally have a group of genes with the same function. The fruit fly is small, has a low cost of rearing and is easy to manipulate in the laboratory. It has short generation time of 10 days at 25°C, a relatively short lifespan and produces a large number of eggs which boosts statistical relevance of the data obtained. *Drosophila* shows complex behavioral phenotypes including social aggregation, re-enforced learning as well as sleep activity that help address questions of brain function. Transgenic fly lines can be created using numerous sophisticated genetic and molecular tools such as insertions of P-elements (72) CRISPR, RNAi silencing, tissue specific GAL4-UAS expression system. Additionally, genome-wide genetic screening, genome-wide analyses with deep sequencers, such as RNA-seq and ChIP-seq, and metabolomics analyses allows us to enquire the cellular and molecular mechanisms of aging and age-related diseases. *Drosophila* has been crucial in the discovery and understanding of innate immune signaling and the development of the nervous system. The fly exhibits multiple physiological changes associated with aging and age related diseases such as reduced locomotive ability (73, 74), impaired learning and memory (75), progressive decline in intestinal barrier function (76), increased inflammation (77), reduced reproductive capacity, and altered neuronal function (78, 79). Additionally, several environmental manipulations such as effects of dietary restrictions are easy to observe (80).

The process of development of neurons is conserved from flies to humans. *Drosophila* has a relatively complex nervous system that is separated from the rest of the body with the blood brain barrier built by glial cells and neurons (81). It's CNS contains about 200,000–300,000 neurons can be histologically divided into two distinct regions (82): the neuronal cell cortex, formed by all the neuronal cell bodies, and a synapse dense neuropil, to which all the dendrites and axons project (83). The fly brain is a sophisticated structure that has several sub-structures: including the antennal lobes, the mushroom bodies, the central body complex, the protocerebrum, the optic lobes, the posterior slope, and lateral deutocerebrum. Sensory organs and the musculature send signals to the CNS via peripheral nerves. The neurons in these associated structures are supported with glial cells. Apart from being the resident immune cells for the CNS, glial cells are responsible for maintaining ionic homeostasis, recycling neurotransmitters, and for the formation of the blood brain barrier (83). *Drosophila* glial cells can be largely categorized on the basis of their location and/or morphology. There are six morphologically and molecularly distinct glial subtypes; perineurial glia (PG), subperineurial glia (SPG), cortex glia (CG), ensheathing glia (EG), astrocytes-like glia(ALG), and wrapping glia (EG in the PNS) (84). The surface of CNS and the peripheral nerves are covered with a thick carbohydrate-rich lamella secreted by perineural glia (PG) and macrophages (85). This PG layer is discontinuous and forms glia–glia pleated septate junctions (pSJs) with subperineural glial cells (SPG) that lie directly below the PG layer. These cells establish the Blood Brain Barrier (BBB) and separate the neuronal elements from the potassium-rich hemolymph. Apart from the BBB, the peripheral nerves have a specialized form of ensheathing glia called wrapping glia, that encloses motor and sensory axons (86). Deeper in the CNS beneath the BBB lie cortex glia, ensheathing glia, and astrocytes-like glia which are closely associated to neurons (87). Cortex glia or cell body associated glia are found within the cell cortex and invade the space between neuronal cell bodies. These cells are in contact with the tracheas and the BBB, suggesting that they are likely responsible to transfer nutrients and gases from the hemolymph to neurons (81). The ensheathing glial cells compartmentalize the brain by forming a sheath around the neuropil (88). These cells are responsible for the phagocytoses of axonal debris (89). As the name suggests astrocyte-like glial cells are functionally and morphologically similar to mammalian astrocytes (90). They are responsible for the maintenance of neurotransmitter homeostasis and in regulating circadian rhythm (91).

Additionally, a large amount of effort has been exerted in creating many distinct *Drosophila* models for a range of neurological disorders such as Parkinson's disease (PD) (92), Alzheimer's disease (AD) (93, 94), and polyglutamine diseases (polyQ) (95, 96). Many of these diseases are caused by abnormal production or accumulation of different proteins such as the accumulation of Lewy bodies in PD, amyloid plaques in AD, and inclusions in polyQ diseases. These protein defects are not normally observed in *Drosophila*. However, they can be artificially produced in flies by introducing human genes into the genome and over expressing them in neurons through the UAS/GAL4 system (97). This is both an advantage and a disadvantage. This technique helps replicate human-like morphological lesions of these diseases and devise screens to genetically identify mutations that suppress the extend of the resulting lesions. However, it is difficult to distinguish the immune responses to such protein build-up from mere non-specific stress responses due to overproduction of an exogenous protein (98).

## Introduction to *Drosophila* Immunity

The innate immune system, an immune reaction with broad specificity, is an organism's first line of defense. It is centered on receptors, which target conserved features of microbial invaders and expeditiously activate downstream cascade to destroy pathogens (99). In jawed vertebrates and some jawless fishes (lampreys) this activation leads also to the induction of adaptive immunity. Unlike those vertebrate categories however, insects lack an adaptive immune system and therefore rely on a relatively sophisticated set of innate defense responses for their survival. The development and function of these reactions are shown to be shared with higher organisms and can be used to study innate immunity and inflammation in humans (100).

Due to the wide range of genetic manipulation techniques it offers, *Drosophila* has been a powerful model to study innate immunity (101). It utilizes a wide range of actions to form effective barriers against pathogens, first of which is a local immune response. This includes the elimination of incoming pathogens by constitutive secretion of AMPs and by reactive oxygen species (ROS) in barrier epithelia such as gut, genitals, cuticle (102, 103), followed by a cellular response which includes engulfment, entrapment, and melanization of the pathogen (104–106). The final response is marked by the rapid synthesis of antimicrobial peptides (AMPs) in the haemocytes and the fat body. The AMPs are regulated by two signaling pathways: The Toll pathway, which was the first in the family of Toll-like receptors discovered in a wide range of organisms from sea urchins to humans, and the IMD pathway homologous to the tumor necrosis factor receptor 1 (TNFR1) in mammals (107).

**Figure 1:**
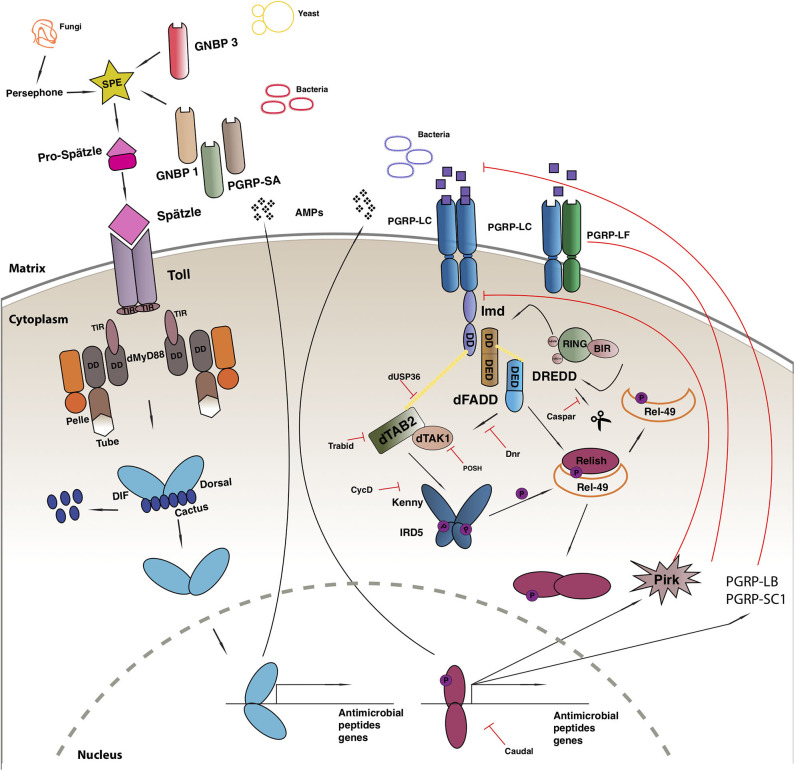
*Drosophila* immune response. Toll pathway (on left) and IMD pathway (on right). See main text for details to the legend.

The Toll-mediated responses are triggered by bacterial or fungal infection which leads to the activation of two Rel transcription factors, Dif and Dorsal that regulate hundreds of genes [reviewed in (100)] (Figure 1). Apart from its role in immunity, the Toll pathway plays a crucial role in the determination of the dorsal-ventral polarity during *Drosophila* early embryogenesis (108, 109). In order to initiate the Toll response, bacterial or fungal pathogens, are sensed by receptors in the form of Peptidoglycan Recognition Proteins (PGRPs; in this case PGRP-SA) or Glucan Binding Proteins (GNBP1, GNBP3) or through cleavage of endogenous proteases (such as Persephone or Psh) (110–112). The next step gives rise to an extracellular proteolytic cascade that culminates in the proteolytic cleavage and activation of the Toll receptor ligand Spatzle (Spz) leading to its activation (113). Spatzle binds to the Toll receptor that recruits MyD88 through its TIR domain, which further interacts with Tube and Pelle through their respective death domains (DD) and promote the phosphorylation of Cactus. Cactus is the *Drosophila* IkB homolog and is bound to Dorsal and/or Dif, inhibiting their activity and nuclear localization. Once degraded, Dorsal and Dif translocates to the nucleus and ultimately leads to the transcription of AMPs and other target genes [reviewed in (114)].

Another evolutionarily conserved singling cascade is the immune deficiency (Imd) pathway (Figure 1), the activation of which is achieved with the help of two PGRP receptors namely, PGRP-LC and PCRP-LE. These receptors bind better to DAP-type peptidoglycan present on Gram-negative bacteria and Gram-positive bacilli and trigger a response, that finally leads to the activation of Rel/NF-κB transcription factor Relish (115). The activated PGRP receptors initiates a signaling cascade and recruits a protein complex containing Imd, a death domain protein, dFadd, and Dredd. Dredd is the caspase-8 homolog, is activated by Iap2 (inhibitor of apoptosis 2) and cleaves Imd to reveal a site important for its role as a transient signaling hub. Iap2 then ubiquitnates Imd and recruits Tab2/Tak1 complex to the reaction site that phosphorylates the Drosophila IKK complex (116). Relish is activated by two events: the phosphorylation of its *N* terminal by Tab2/Tak1 complex and the cleavage of its *C* terminal ANK repeats domain by Dredd. The activated *N* terminal (Rel-68) enters the nucleus and initiates transcription of AMPs (116).

The improper activation of the immune signaling pathways is associated with inflammation, cancer, and neurodegeneration (77, 117), and leads to developmental defects during ontogenesis (118–120). To prevent the harmful consequences of unwarranted activation, the pathway is firmly regulated by extracellular and intracellular proteins.

### Negative Regulation of IMD

The IMD immune response is tightly regulated at many levels, first of which is the dilution of the activating signal. This is done by the secreted PGRP-SC and PGRP—LB amidase that breakdown bacterial peptidoglycan into non-stimulatory fragments in the extracellular matrix (119, 121). On the plasma membrane, the three PGRP-LC isomers interact with each other to suppress spontaneous dimerization and reduce the number of functioning receptors (122). Additionally, PGRP-LF binds with PGRP-LC to form non-signaling heterodimers and down regulate the response (123). Intracellularly, PIMS/pirk/rudra coimmunoprecipitates with Imd causing a disruption in its association with the cytoplasmic tail of PGRP-LC receptor leading to its the depletion from the membrane. *Pirk* mutant have constitutively activated immune response and are short lived. Moreover, flies over expressing pirk have a reduced Imd response (124–126). Dnr1 inhibits the activity of Dredd caspase by promoting its proteolytic degradation. *Dnr 1* mutants have shorter lifespans and exhibit age-dependent neuropathology (127). The activity of the Dredd caspase is also impaired by Caspar, which inhibit Dredd-dependent modification of Relish and further blocking its translocation to the nucleus (13). The activity of NF-kB is regulated by several ubiquitin-mediated interactions, deregulation of these factors cause chronic inflammation and cancer (128). Further negative regulators of the Imd pathway include SkpA, dUSP36, CYLD, POSH, Trabid, and transglutaminase (TG). *Drosophila* ubiquitin-specific protease 36 (dUSP36) inhibits the K63-polyubiquitinated Imd build up and promotes its degradation (129). It is a negative regulator of the pathways as silencing *dUSP36* constitutively activates the IMD signaling pathway. This activation is lost in germ free flies leading to the hypothesis that this interaction might be microbiome dependent (130). K63-linked ubiquitination of dTAK1 is monitored by another negative regulator of IMD known as Trabid. Flies lacking this protein show a remarkable increase in the amount of the IMD target, Diptericin with a dramatic reduction of the lifespan (131). A further negative regulator of TAK1, POSH (Plenty-of-SH3s) prevents engagement with the JNK scaffold (132). Downstream, the cylidromatosis disease homolog dCYLD interacts with the *Drosophila* IKKγ homolog Kenny and disrupts downstream signaling (133). In its absence, triglyceride and AMP levels increase (133). At the level of Relish finally, SkpA and the proteasome-ubiquitin pathway (134) and Transglutaminase (135) suppress NF-κB activity.

## Predisposition to an Overactive Immunity Causes Neurodegeneration

Human genetics and animal model research have illustrated the correlation between innate immunity in the brain and the pathogenesis of neurodegenerative disorders (Table 1). Increasing amount of evidence suggests that the accumulation of aggregated proteins is only a part of the pathology of neurodegenerative disorders and not the full story [reviewed in (143)]. Increasing evidence suggests the role of the immune system as an aetiological mechanism that influences not only the pathology of the diseases but also modulates basal levels of age dependent neurodegeneration in the context of healthy aging (144).

**Table 1:** Summary of papers at the junction of immunity and neurodegeneration in *Drosophila*.

**Gene**	**Immune phenotype**	**Neurological phenotype**	**References**
*pirk*/*rudra*/*pims* (loss of function)	Overactivation of IMD	Locomotion, reduced lifespan, brain neurodegeneration	(77)
*trabid* (loss of function)	Overactivation of IMD	Locomotion, reduced lifespan, brain neurodegeneration	(77)
*transglutaminase* (loss of function)	Overactivation of IMD	Locomotion, reduced lifespan, brain neurodegeneration	(77)
*dnr1* (loss of function)	Overactivation of IMD	Locomotion, reduced lifespan, brain neurodegeneration	(127)
*cdk5* (loss of function)	Overactive immunity through reduction of autophagy	Loss of DA neurons	(19)
Toll and IMD activity increases	Overactive immunity	Age-dependent neurodegeneration in a model of pTBI	(136)
*yorkie* activity	Suppression of IMD and Toll	Reduction of PolyQ-mediated neurodegeneration	(137)
*relish* (knock-down in DA neurons)	Suppression of IMD	Increased resistance to paraquat, rescue of motility defects and DA neurons in a *Drosophila* model of PD	(138)
*relish* (loss of function)	Suppression of IMD	Suppression of retinal degeneration in norpA mutants	(139)
*draper* activity	Glial phagocytosis	Better clearance of Aβ-amyloid	(140)
*draper* (age-dependent reduction)	Dysfunctional glial-mediated engulfment	Neuronal death	(141)
*spz-5* (in neurons)	Activation of Toll-6 in glia	Neuronal death; dying neurons signal to glia	(142)

*We only list those showing a causative link between immune activity or immune-related signaling and neurological phenotype*.

### Loss of Negative Regulation of IMD

Cao et al. illustrated that chronic activation of the immune response in the wild type *Drosophila* brains causes neurodegeneration (127). Flies with loss of function mutations in a Relish repressor gene *dnr-1* show signs of early neurodegeneration and an increase in the number of Relish target genes transcripts in the fly brain. The authors suggest the cause of this neurodegeneration as AMP-associated toxicity caused by constitutive expression or an extremely high level of AMPs present in neurons or glial cells. The study also revealed that the overexpression of AMPs in nervous tissue can cause neurodegeneration and established a causative relationship between neurodegeneration and IMD signaling. However, overexpression of AMPs brings expression to much higher levels than the *dnr-1* mutant and therefore more work is needed to prove this point. Nevertheless, the possibility of a neuroprotective role of negative regulators in neuronal viability is clearly suggested here (127).

A similar result were obtained by Kounatidis et al., who demonstrated an age-dependent increase in NF-kB- controlled immune activity in *Drosophila* in the context of healthy aging (77). Most of it was dependent on the microbiota, as germ free flies had much reduced age-dependent AMP increase compared to conventionally reared insects (77). Nevertheless, there was consistently a 2–4x age-dependent AMP increase in germ free flies as was a clear sterile inflammation in the brain. Moreover, the loss of Trabid, Pirk, and TG in neuronal tissue resulted in shortening of lifespan, locomotion defects, and the formation of brain lesions. This phenotype was rescued once Relish was suppressed in glial cells of these flies. In wild type flies, suppressing *relish* in glial cells resulted in lifespan extension. Therefore, genetic predisposition to higher immune levels with mutation in *trbd, pirk*, and *tg* led to early neurodegeneration and curtailed lifespan (77).

### Autophagy and Immunity

An interesting connection between autophagy, immunity and neurodegeneration was recently made by the observation that mutants for the Cdk5 protein kinase have increased AMP expression in the brain and loss of dopaminergic neurons. This happens because loss of Cdk5 disrupts autophagy and this results in increased levels of immunity (19). This point is important since autophagy seems to be necessary and sufficient to drive the increase in AMP levels. Given the dysregulation of Cdk5 and innate immunity in human neurodegeneration and the conserved role of this kinase in the regulation of autophagy, this sequence of events is likely to resemble what happens in humans (19). However, the connection between immunity and autophagy remains largely unexplored. One indication is the interaction between Kenny (IKKγ) and the autophagy protein Atg8, which targets Kenny for selective degradation. Loss of Atg8, “releases” Kenny, enhancing IKK signaling, and resulting in chronic IMD induction (145).

### Neurodegenerative Disease Models

The Penetrating traumatic brain injury (pTBI) model show a greater expression of AMP genes and an over activation of the innate immune response in both young and older flies (136). The positive interaction between pTBI and aging was further supported by the high expression of Imd negative regulators in older pTBI fly brains. The study indicated that aging exasperates the immune response caused by pTBI and causes neurodegeneration. Additionally Yorkie, a co-activator of Hippo pathway was also shown to reduce polyglutamine (PolyQ)-mediated neurodegeneration by negatively regulating Toll and Imd pathways *via cactus* and *relish*, respectively (137).

Recently, transcription of innate immune genes were observed as the prominent response to paraquat in a *Drosophila* model of PD (138). Interestingly, Relish knock down in dopaminergic neurons conferred resistance to paraquat and rescued both motility defects and loss of dopaminergic neurons. The study indicates that the immune reaction might not be protective and indicate potential drug targets for preventing neuronal loss during PD. Immunity induced neurodegeneration can explain the neurodegenerative phenotypes observed in both *ataxia–telangiectasia mutated (ATM)* gene and retinal degeneration in *norpA* (no receptor potential) mutants (139). Reduction in the ATM kinase activity in the glial cells may be responsible for the increased innate immune response through protein phosphorylation and cause neurodegeneration in these mutants. Furthermore, retinal degeneration in *norpA* mutant flies was shown to be dependent on Relish and Dredd.

### Neuroprotective Roles of Immunity

In addition to the role of the long-term heightened IMD signaling in causing neurodegeneration, there is also a neuroprotective aspect of glial signaling components connected to immunity. Ray et al. showed the neuroprotective role of the glial engulfment receptor, Draper, in *Drosophila* model of AD (140). Overexpression of glial *draper* reverses amyloid (Aβ) accumulation along with AD associated behavior phenotypes. They also show that protein degradation pathways are expressed downstream to Draper in response to amyloid accumulation. This supports the theory that glial cells may be responsible for the clearance of neurotoxic amyloid peptides in the brain through a Draper/JNK/STAT92E signaling cascade (140). Draper is also observed to have a significant role in clearance of damaged axons. Purice et al. observed an age-dependent decline in the levels of Draper that causes dysfunctional glial engulfment in older flies (141). Dying neurons activate Toll receptor ligand, Spz, in the cortex glia, that further drives the expression of Draper to ensure efficient clearance of the neuron (142).

### Gut-Brain Axis

Recent studies have also focused on the role of gut- brain crosstalk and neurodegeneration. Wu et al. highlighted the effect of enteric infection in AD progression (146). Gut dysbiosis in AD mutant flies caused an increase in haemocyte recruitment to the brain and activation of TNF-JNK mediated neurodegeneration. Neurodegeneration and reduction in lifespan were rescued in flies with genetically depleted Eiger (an activator for JNK pathway) in the brain, further supporting the hypothesis. Westfall et al. explored how symbiotic and probiotic formulation can influence gut brain signaling and delay the progression of AD (147).

### Limitations

Needless to say, no model system is without limitations. It is important to note that like most invertebrate organisms, the fruit fly is evolutionarily distant from humans and does not accurately mimic all the neurodegenerative phenotypes observed in human diseases such as tau aggregates and plaques (148). *Drosophila* lacks an adaptive immune response making it difficult to recapitulate complex changes in the immune response, that might take place during aging. Additionally, the hemolymph of the fly contains primitive hemocytes which cannot undergo DNA rearrangement and somatic hypermutation like mammalian lymphocytes. Unlike mammals, flies do not possess microglia rather, all glial cells can perform microglial tasks; such as engulfing neuronal corpses during development (84). However, this restricts studies that attempt to understand the complicated relationship between the immune system and the relation between neuroprotection and neurodegeneration. Nevertheless, the studies summarized here highlight evidence suggesting that the immune system plays an important role in neurodegenerative disorder in *Drosophila*. Two key contributors to lifespan reduction and neuropathy are overproduction of AMPs and impaired phagocytosis. Even though animal models do not represent the diseases completely (for example in the lack of direct orthologs for the human proteins prone to aggregation in AD or PD), comparative studies of brain development and the innate immune response have demonstrated significant evolutionary conserved mechanisms between vertebrates and invertebrates. Moreover, the deregulation of innate immunity as etiology for neurodegeneration stands in *Drosophila* even in the absence of tau or β-amyloid. There is a large therapeutic potential of immunomodulation and therapeutic immunization (149) to help combat the development of such diseases by screening fast in whole animal models such as the fly. Moreover, since the role of immune activity in microglia and astrocytes in neurodegeneration is well-documented, we could envisage that negative regulators of immunity could be potential candidates for early interventions.

## Conclusion

The precise mechanism of the development of neurodegenerative diseases is still unknown and this presents a challenge for the development of treatments and therapies. Currently, therapies focus only on treating isolating disease symptoms such as protein accumulation, sleep disturbances, memory loss, or behavioral changes. Additionally, disease modifying therapies are largely unsuccessful and there is need for more drug candidates to enter the pipeline. Since most of the cases of neurodegeneration are only diagnosed after severe neuronal loss. Exploring preclinical symptoms as potential therapy can facilitate the development of treatments for the early symptoms of the disease. Aberrant immune regulation resulting in chronic inflammation long before neurological symptoms manifest themselves may be at the root of these diseases. We believe that *Drosophila* represents an ideal compromise between its relevance to humans and its demographic power and genetic tractability, making it a model of choice for understanding mechanistic aspects of age-related neurodegeneration.

## Author Contributions

SA wrote the first draft and drew the figures. PL worked on subsequent drafts with SA. All authors contributed to the article and approved the submitted version.

## Conflict of Interest

The authors declare that the research was conducted in the absence of any commercial or financial relationships that could be construed as a potential conflict of interest.
